# Analyzing the Effect of Weak External Transcranial Magnetic Stimulation on the Primary Dominant Frequencies of Alzheimer Patients Brain by Using MEG Recordings

**DOI:** 10.3390/medicina57111164

**Published:** 2021-10-26

**Authors:** Photios Anninos, Adam Adamopoulos, Nikolia Anninou, Nikolaos Tsagas

**Affiliations:** 1 Laboratory of Medical Physics, Department of Medicine, Democritus University of Thrace, 68100 Alexandroupolis, Greece; adam@med.duth.gr; 2 Private Sector Veterinarian, 68100 Alexandroupolis, Greece; nianninou9@gmail.com; 3 Department of Electrical Engineering, Democritus University of Thrace, 7100 Xanthi, Greece; ni.tsagas@gmail.com

**Keywords:** Alzheimer Disease, magnetoengephalography, pico-Tesla transcranial magnetic stimulation

## Abstract

*Backround and Objectives:* Alternative, non-invasive, and non-pharmaceutical options are gaining place in the battle of Alzheimer’s Disease treatment control. Lately, the magnetic stimulation of the brain is the most prevalent technique with encouraging results. The aim of this study is to establish any possible change on the Primary Dominant Frequencies (PDF) (range 2–7 Hz) of the affected brain regions in Alzheimer Disease (AD) patients after applying extremely weak Transcranial Magnetic Stimulation. *Materials and Methods*: For this purpose, all AD patients were scanned with the use of MagnetoEncephaloGraphy (MEG) recordings through a whole-head 122–channel MEG system. *Results:* Our results exerted statistically significant PDF changes due to weak TMS accompanied by rabid attenuation of clinical symptoms. *Conclusion:* Thus, this is the first time that a positive therapeutic effect is being demonstrated even at pico-Tesla range magnetic fields in a small clinical group of studies for AD.

## 1. Introduction

Alzheimer Disease (AD), one of the most prevailing types of dementia, is a neurodegenerative disorder with a worldwide occurrence, which is strongly related to the aging process [1]. The age by which the AD appears determines its characteristic form [1,2]. Its clinical phenotype is characterized by progressive memory disturbances, hallucinations, speaking or communication difficulties, and orientation as well as motor disorders [2,3,4].

Numerous histopathalogical modifications take place in the AD patients’ brain that eventually lead to general brain atrophy mainly in the temporal and frontal lobes [5,6,7,8]. The senile plaques, the neurofibrillary tangles, and the early degeneration or death of neurons are indicative biomarkers of the AD [5].

Several studies have already been conducted in order to provide a full-spectrum theory of AD’s pathogenesis [9]. Complex biochemical, molecular, and cellular abnormalities, together with multiple gene mutations inducing neurodysfunction and neurodegeneration, are involved in Alzheimer’s manifestation [9].

Despite the progress in research, providing insight into the pathophysiology of AD, the therapeutic options of dementia syndrome still remain a challenge. Transcranial Magnetic Stimulation, as many studies imply, can positively modify brain activity in AD and thus, mitigate clinical symptoms [10]. The brain stimulation frequencies mainly used up to now are >3 Hz with the effect on AD profile being depended on various factors and parameters [10].

In our clinical trial, in order to estimate the atypical magnetic fields, due to abnormal neuronal activity of the AD patients’ brain, we used the sensitive technique of the Transcranial MagnetoEncephaloGraphy (T-MEG) [11,12]. This magnetic field of the brain is the result of the cellular ionic activity due to the dynamical alterations of the neuron membrane potentials. It is very weak, approximately pT = 10^−12^ T, and can easily be detected and scored by using MEG recordings [13].

Our goal is to detect possible alterations in the front temporal lobes of the patient’s AD brain through the use of pT-TMS electronic device after applying FFT analysis [14]. This device is a 122-coil helmet, arranged in five groups disposition, which covers the main 7 brain areas of the patient (Table 1) and is designed to create pT-TMS range modulations of magnetic flux in the alpha frequency range (8–13 Hz) for every patient [14]. The pT-TMS device format is to produce a square wave equivalent to the neuron’s interaction or communication in the brain [15,16,17,18,19,20].

## 2. Materials and Methods

### 2.1. Sample

The study sample included 10 volunteer patients, 7 male, and 3 female. All the patients that were referred to our laboratory of Medical Physics by neurologists, were diagnosed with AD and had normal complete serum biochemical profile. The patients’ age scale in the present study was from 55 to 72 years.

All AD patients prior the procedure, were informed and gave their consent for the methodology and the aim of the study.

The Research Committee of Democritus University of Thrace gave its approval for the protocol of our study.

The General Secretariat of Research and Technology, GR, and the ERGO AEBE INC, GR (Grant Number: 80623) funded the research.

### 2.2. Procedure

The study was planned for 3 days duration. The first day (day 1) every AD patient underwent, ensuring alertness, a 2 min brain scannin2g session, through the use of the whole head 122-channel 2nd order gradiometer device NEUROMAG-122 SQUID (Neuromag-122, Neuromag Ltd., Helsinki, Finland), and thus, MEG signals were recorded for each subject before pT-TMS. The whole-head 122 channel MEG system, embodied in a specially designed room in order to diminish the magnetic noise and provide accurate results, has already been described in our previous publications [15,16,17,18,19,20].

The second day (day 2) pT-TMS was applied for 60 s on each participant with a whole head device, especially designed to adjust in the Alpha Brain Frequency of every subject (customized brain stimulation). [16,17,18,19,20]. Afterward, a 2 min post-stimulus MEG recording was performed. The same procedure was repeated on the third day (day 3) with the presence of neurologists, thus as to perform a clinical and neuropsychological examination of the group.

### 2.3. Data Acquisition

**Figure 1:**
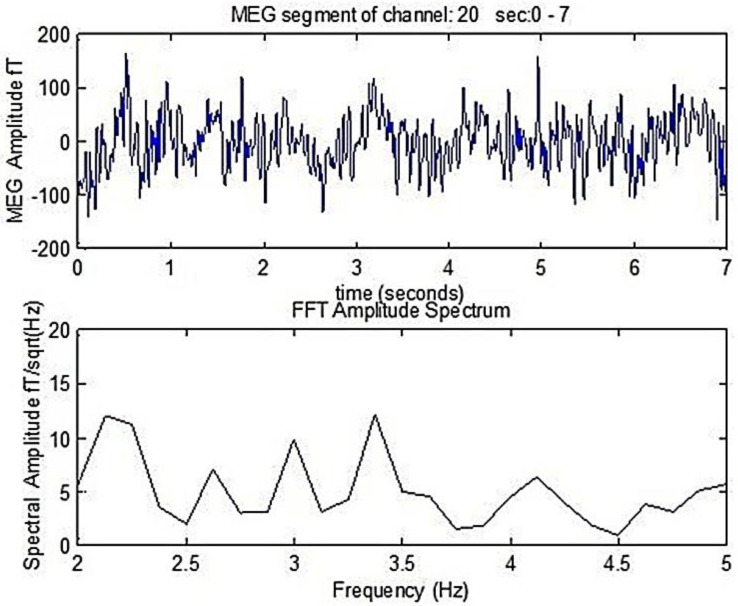
A 0–7s MEG segment of an AD patient (up) and the corresponding amplitude spectrum that is obtained after the application of FFT (bottom). The dominant frequency is 2.1 Hz.

In order to score the Primary Dominant Frequencies (PDF) changes, we developed a software program [15,16,17,18,19,20], which detects the PDF of the power spectra of the MEG obtained from AD patients affected brain region prior and following weak TMS. The primary frequency of each signal was detected after applying Fast Fourier Transform (FFT), as is shown in Figure 1. There a 7 s duration MEG segment of an AD patient is shown, as well as the corresponding amplitude spectrum that is derived following the FFT analysis. The primary dominant frequency in the amplitude spectrum shown in Figure 1 is 2.1 Hz.

Two-dimensional color maps for the spatial distribution of the primary dominant frequencies were constructed based on these frequencies estimation for each affected brain region and channel. Each color in the maps depicts different primary dominant frequencies. Finally, each number in the map squares indicates groups of MEG channels, in line with the whole-head device for each brain area according to Table 1.

### 2.4. Statistical Analysis

Data were displayed as mean ± standard error of the mean. Single comparisons were performed with unpaired Student’s *t*-test. Statistical analysis was employed by SPSS 16.0 (SPSS Inc., Chicago, IL, USA). Values with *p* < 0.05 were considered statistically significant.

## 3. Results

In Figure 1, through compendious analysis, we identified the magnitude of the primary dominant frequency (MPFD) of the amplitude spectra after pT-TMS and FFT analysis.

In Table 1, we present the brain regions and the respective channels in each brain region. In Table 2 are displayed possible modification effects before and after the use of pT-TMS on each AD patients’ brain. In particular, the maximum frequency is shown between the first MEG recording before stimulation (BS) and the MEG recording after stimulation (AS) for the affected brain regions of the 10 AD patients.

In Table 3 is shown the statistical analysis for the AD patients using an unpaired *t*-test. The results were statistically significant at 7 out of 10 patients (70%).

In Table 4 is shown the AD patient’s symptomatology before and after pT-TMS effect, as they were evaluated by interviews by clinicians. We observed that 3 out 10 AD patients did not show improvement (30%) namely (nr. 2, 4, 8), according to the statistical analysis of Table 3. Especially, 1 out of 3 female AD patients (33%) and 2 out of 7 male patients (28%) did not show improvement.

Finally, in Figure 2, Figure 3, Figure 4, Figure 5, Figure 6, Figure 7, Figure 8, Figure 9, Figure 10 and Figure 11, color maps depict frequencies alteration before and after magnetic stimulation of each AD patient.

## 4. Discussion

AD is a common degenerative brain disorder with multi-complex clinical symptomatology, which debilitates the patient from daily routine and reduces the quality of life [21,22]. Despite the fact that numerous studies have been made involving Alzheimer’s pathogenesis, still there are many blur paths that need to be clarified [23]. Probably this is the reason why we are still not talking about a completely efficient cure.

As far as we know, the therapeutic strategies for AD preserve two ways, the pharmaceutical and the non-pharmaceutical trail [22]. Drugs approved for Alzheimer’s management emphasize on the Aβ brain plaques accumulation control or focus on cell signal processing with the clinical benefits being semi-promising [23]. Transcranial Magnetic Stimulation, on the other hand, overrules among the alternative treatment options [24,25]. Up to now, clinical trials that have been working on high frequencies brain stimulation >3 Hz and mainly at the range of 10 to 20 Hz, rather than low frequencies (≤1 Hz), provide positive results several weeks or months post-TMS [24,25].

In our study, 3 out of 10 participants with extremely serious symptoms did not have any improvement, through weak TMS, in their clinical profile. Our results are in line with previous studies applying a range stimulation protocol from 1 Hz up to 20 Hz on patients with mainly severe symptomatology and prolonged disease progression [25].

On the other hand, the pT-TMS on AD patients with mild symptomatology exhibited a positively statistically significant effect since 7 out of 10 patients declared substantial clinical improvement. To our knowledge, this is the first time that TMS, as weak as 10^−12^ Tesla, is applied on AD patients, through a whole head personalized brain stimulation apparatus, for a very short period of time (2 sessions of 60 s each, 24 h apart) and accompanied by MEG neurosensitive scanning, pre- and post-stimulus.

However, the encouraging outcome with the up to now AD knowledge cannot be totally explicated. Still, one rational explanation might be the fact that weak magnetic fields hold a strong positive impact on the pineal gland’s ability, through melatonin, to control endogenous opioid signal pathways [26,27,28] and several neurotransmission nets [29,30], which show significant changes in cognitive disorders. Additionally, another perspective is that this type of magnetic stimulation is capable of modulating neurons cell membrane’s overall biological profile and role in the neurotransmission process, which is significantly impaired in AD patients [31].

## 5. Conclusions

To summarize, the use of pT-TMS preserves a promising positive outcome of the clinical image of AD patients, as it lightens’ their daily symptoms, in no time, with the use of a totally safe model. Nevertheless, in order to prove pT-TMS effectiveness on AD treatment, further studies with larger groups of AD patients are needed to be conducted.

## 6. Patents

Anninos, P.A, and Tsagas, N. Electronic apparatus for treating Epileptic individuals. USA patent 5453072, 1995.

## Figures and Tables

**Figure 2:**
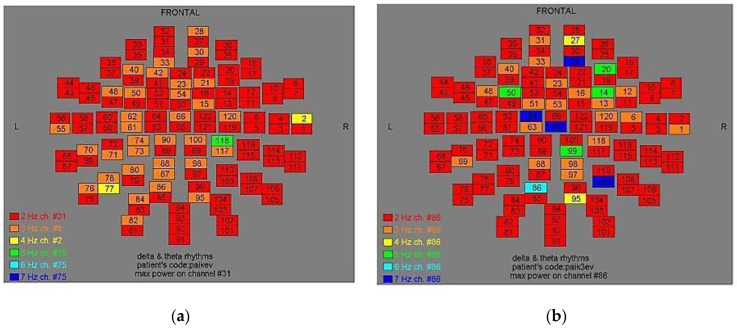
Dominant frequency color mapping for patient #1, before (**a**) and after (**b**) magnetic stimulation.

**Figure 3:**
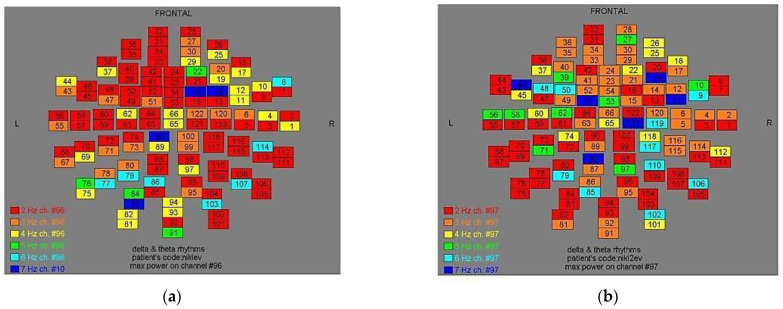
Dominant frequency color mapping for patient #2, before (**a**) and after (**b**) magnetic stimulation.

**Figure 4:**
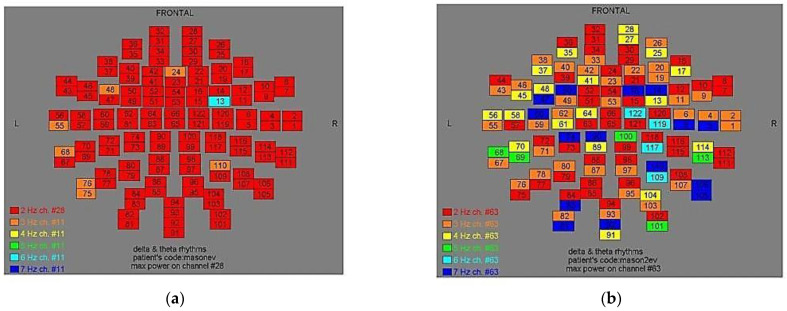
Dominant frequency color mapping for patient #3, before (**a**) and after (**b**) magnetic stimulation.

**Figure 5:**
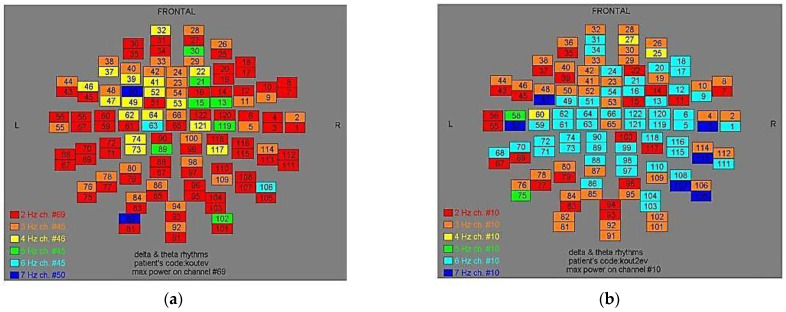
Dominant frequency color mapping for patient #4, before (**a**) and after (**b**) magnetic stimulation.

**Figure 6:**
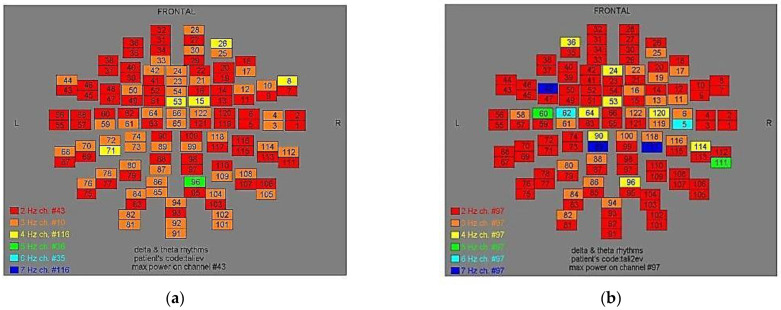
Dominant frequency color mapping for patient #5, before (**a**) and after (**b**) magnetic stimulation.

**Figure 7:**
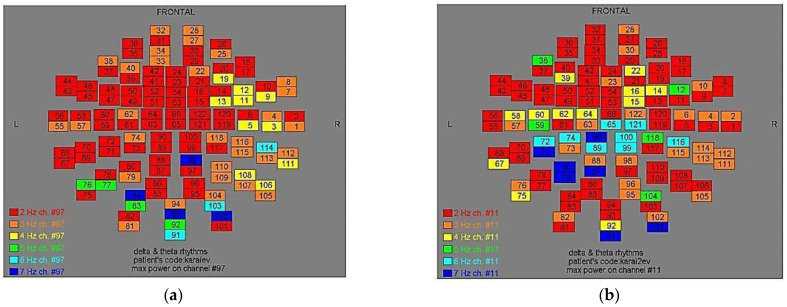
Dominant frequency color mapping for patient #6, before (**a**) and after (**b**) magnetic stimulation.

**Figure 8:**
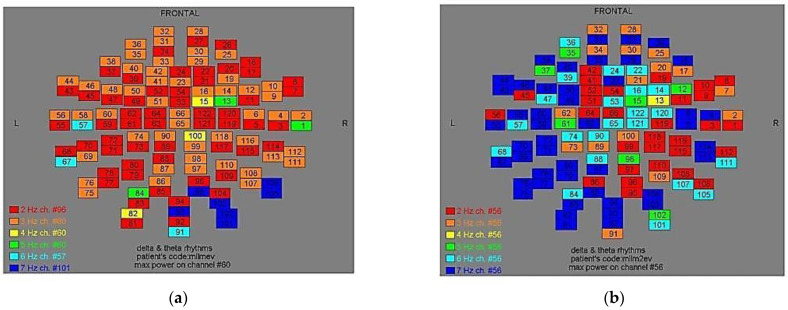
Dominant frequency color mapping for patient #7, before (**a**) and after (**b**) magnetic stimulation.

**Figure 9:**
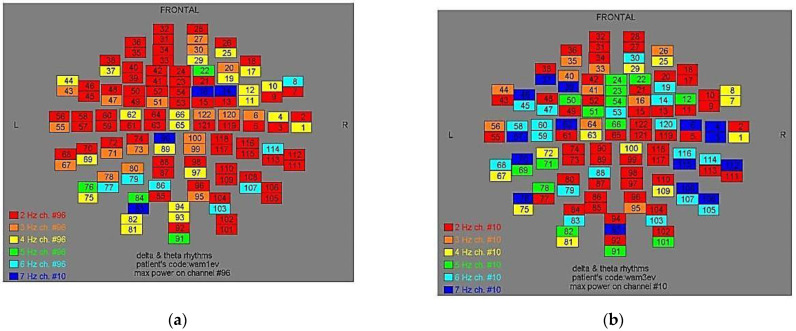
Dominant frequency color mapping for patient #8, before (**a**) and after (**b**) magnetic stimulation.

**Figure 10:**
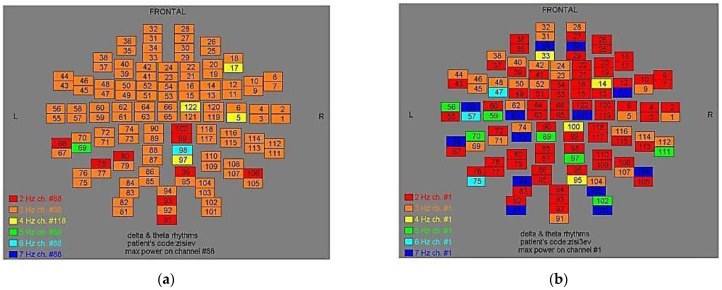
Dominant frequency color mapping for patient #9, before (**a**) and after (**b**) magnetic stimulation.

**Figure 11:**
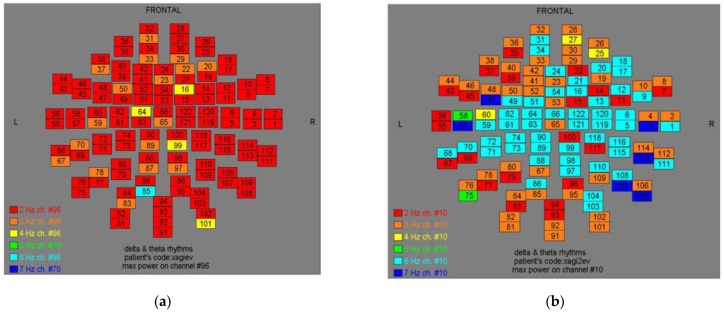
Dominant frequency color mapping for patient #10, before (**a**) and after (**b**) magnetic stimulation.

**Table 1:** Brain areas and the relative channels in each brain area.

Main Brain Areas	Channels
Right Temporal	1–14, 111–120
Left Temporal	43–50, 55–62, 67–74
Right Parietal	5–6, 11–16, 97–100, 109,110, 115–122
Left Parietal	
Frontal	17–42
Occipital	75–86, 91–96, 101–110
Vertex	13–16, 49–54, 61–66, 73, 74, 89, 90, 99, 100, 117–122

**Table 2:** Maximum frequency between the first MEG recording before stimulation (BS) and the MEG recording after stimulation (AS) for the affected brain regions of the AD patients.

P	RT BS	RT AS	LT BS	LT AS	RP BS	RP AS	LP BS	LP AS	F BS	F AS	Figures
1	5	5	3	5	5	6	3	6	3	6	Figure 2
2	6	6	4	7	6	6	4	7	5	5	Figure 3
3	6	7	3	7	6	7	3	7	3	4	Figure 4
4	5	7	7	7	5	6	7	7	5	6	Figure 5
5	4	7	4	7	4	7	4	7	4	4	Figure 6
6	4	6	3	7	7	6	3	7	4	5	Figure 7
7	4	7	4	7	7	7	2	7	3	7	Figure 8
8	7	7	4	7	7	7	7	7	5	7	Figure 9
9	5	7	5	7	6	7	3	7	4	7	Figure 10
10	2	7	3	7	4	6	4	7	3	6	Figure 11

RT: Right Temporal; LT: Left Temporal; RP: Right Peripheral; LP: Left Peripheral; F: Frontal.

**Table 3:** Statistical analysis of the Alzheimer patients’ group. Marked in bold are the statistically significant results (*p* < 0.05).

Patients	Meanf (BS+)	Meanf (AS+)	*t*-Test *p*-Values
1	3.80 ± 1.10	5.60 ± 0.55	**0.0111**
2	5.00 ± 1.00	6.20 ± 0.84	0.0736
3	4.20 ± 1.64	6.40 ± 1.34	**0.0490**
4	5.80 ± 1.10	6.60 ± 0.55	0.1823
5	4.00 ± 0.00	6.40 ± 1.34	**0.0039**
6	4.20 ± 1.84	6.20 ± 0.84	**0.0415**
7	4.40 ± 1.34	6.80 ± 0.45	**0.0053**
8	6.00 ± 1.41	7.00 ± 0.00	0.1525
9	4.60 ± 1.14	7.00 ± 0.00	**0.0015**
10	3.20 ± 0.84	6.60 ± 0.55	**0.0001**

**Table 4:** Clinical symptomatology of the Alzheimer patients as it was assessed by neurologists before and after stimulations (2nd and 3rd day in the lab) (F: Female; M: Male).

Patients	Sex	Symptoms before Stimulation (BS)	Symptoms after Stimulation (AS)
1	M	Memory problems, speaking communications	His symptoms improved
2	F	General atrophy in temporal and frontal lobes and orientation disorders	Her symptoms did not stop
3	M	Memory disturbances, speaking communication	His symptoms improved
4	M	General atrophy in temporal and frontal lobes	His symptoms did not improve
5	F	Memory disturbances orientation difficulties	Her symptoms improved
6	F	Atrophy of temporal and frontal lobes, memory disorders	Her symptoms improved
7	Μ	Difficulties of speaking and memory problems	His symptoms improved
8	Μ	Orientation disorders and memory problems	His symptoms did not improve
9	Μ	Difficulties in memory and speaking	His symptoms improved
10	M	Difficulties in memory and speaking	His symptoms improved

## Data Availability

The datasets used and analyzed during the current study are available from the corresponding author on reasonable request.
